# An Exploration of Adults Transitioning Into Retirements' Perspectives on Vigorous Intermittent Lifestyle Physical Activity

**DOI:** 10.1002/hpja.957

**Published:** 2025-01-07

**Authors:** Bingyan Pang, Joanne A. McVeigh, Craig Thompson, Cecilie Thøgersen‐Ntoumani, Emmanual Stamatakis, Joanna C. Moullin

**Affiliations:** ^1^ Curtin University Bentley Western Australia Australia; ^2^ enAble Institute Curtin University Bentley Western Australia Australia; ^3^ Movement Physiology Laboratory University of Witwatersrand Johannesburg South Africa; ^4^ University of Southern Denmark Odense Denmark; ^5^ University of Sydney Camperdown New South Wales Australia

**Keywords:** health promotion, healthy aging, mixed method, physical activity, retirement

## Abstract

**Issue Addressed:**

Australian adults transitioning into retirement aged 55–75 years (> 50%) do not meet the World Health Organization recommendation of physical activity (PA). One potential strategy to promote PA is through vigorous intermittent lifestyle physical activity (VILPA). This study aimed to investigate barriers and facilitators from adults transitioning to retirement about participation in VILPA and to identify strategies to promote and implement VILPA.

**Methods:**

Thirty adults transitioning to retirement (mean age = 64 years) were recruited to participate in focus groups to provide their perceptions on VILPA. All participants' PAs were measured by accelerometers. A set of semi‐structured questions developed from the findings of a previous scoping review was used to guide focus groups with participants. To increase awareness of PA bouts and intensity, the focus group discussions were prompted by participants' individualised accelerometer‐measured PA reports. The identified barriers and facilitators were mapped to the Theoretical Domains Framework. Intervention strategies were derived from the framework domains.

**Results:**

Three focus groups were conducted. Participants perceived barriers to participation in VILPA stem from health constraints, insufficient awareness about VILPA, and adverse weather conditions. To promote VILPA, adults transitioning to retirement require a better understanding of PA intensities, knowledge of identifying VILPA opportunities, and monitoring and feedback for engagement. Intervention to promote VILPA should include elements of education, persuasion, incentivisation and enablement.

**Conclusions:**

Adults transitioning to retirement perceived VILPA as feasible and convenient for increasing their overall PA.

**So What?:**

The study findings will directly inform the development of a targeted VILPA intervention with key stakeholders and an implementation plan to promote PA in adults transitioning to retirement.

## 1 Background

One in four adults worldwide does not meet the World Health Organization recommendation of physical activity participation (i.e., a minimum of 75 min vigorous‐intensity physical activity or 150 min moderate‐intensity physical activity per week) [1]. In Australia, more than half of adults transitioning into retirement (i.e., reducing work hours and moving toward retirement) aged 55–75 do not participate in sufficient physical activity [2]. Physical activity is a modifiable factor to reduce risks and complications relating to chronic health conditions and all‐cause mortality [1, 3]. Current interventions have had little success in promoting continuous long sessions of physical activity in adults aged 55–75 [4, 5, 6]. Some key barriers to participation in these exercise interventions are lack of time, lack of access to facilities, and cost [4, 7, 8]. Novel options are needed to provide more opportunities to increase physical activity participation.

One potential strategy to promote physical activity participation is through short bouts (< 2 min) of vigorous intermittent lifestyle physical activity (e.g., climbing a few flights of stairs, carrying a small load of shopping) [4, 9]. Epidemiological studies using the UK Biobank accelerometer‐measured physical activity data with > 22 000 individuals (mean age 61.8 years) showed that adults who participated in vigorous intermittent lifestyle physical activity (VILPA) lasting up to one or up to 2 min per bout, three times per day, were shown to have nearly 50% lower risk of cardiovascular disease‐related death, and lower all‐cause mortality than insufficiently physically active adults who did not participate in VILPA [10, 11]. Participation in < 4 min of VILPA per day is also associated with 18% reduction in total incident cancer risk [11]. The objective accelerometer‐measured physical activity data from these studies removed the potential bias in self‐reporting of physical activity participation and allowed a better understanding of the accumulation of short bouts of physical activity throughout the day with little burden to participants. To promote participation in short bouts of physical activity such as VILPA in adults transitioning to retirement, it is essential to understand their behavioural barriers and facilitators.

**FIGURE 1:**
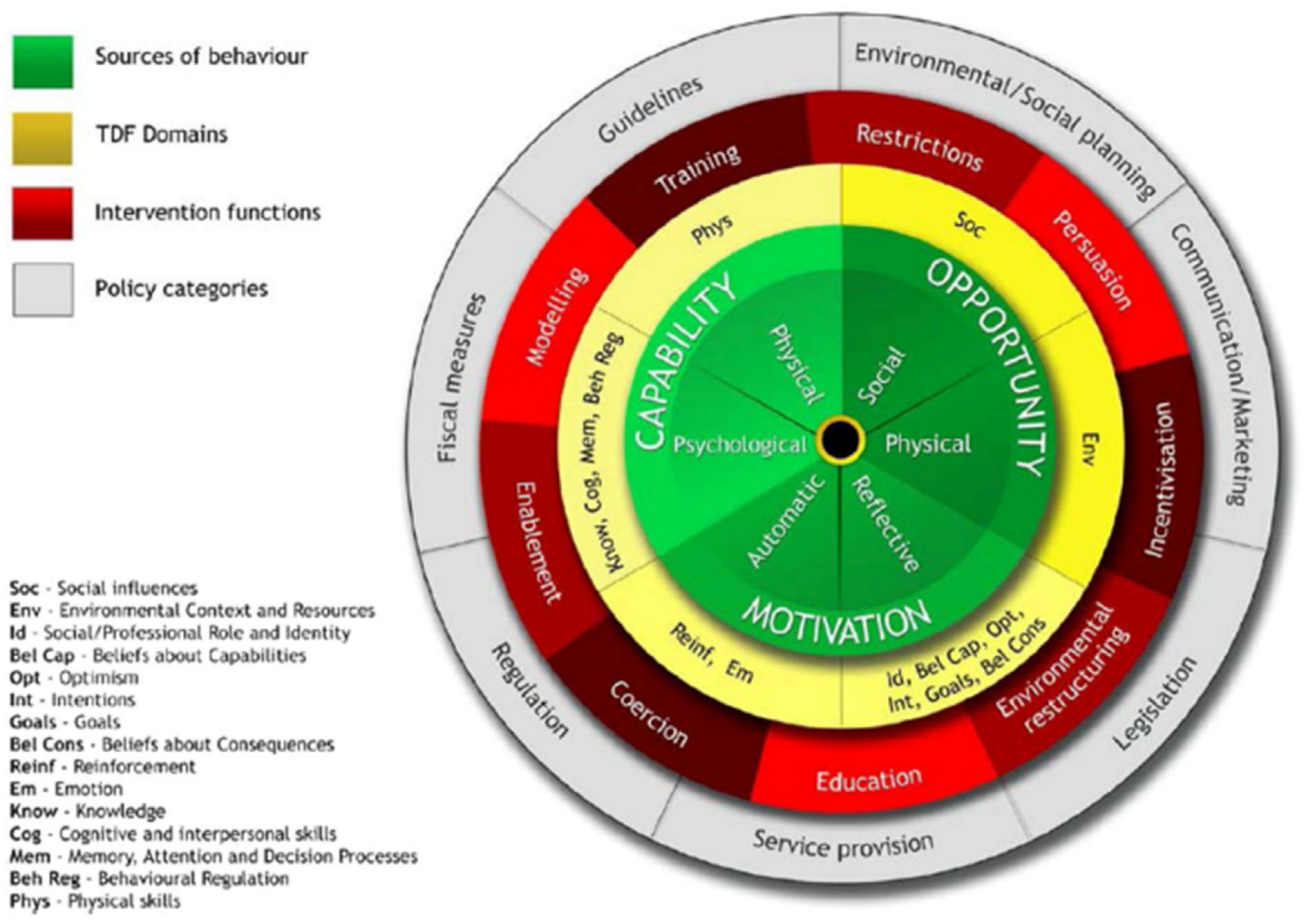
Behaviour Change Wheel linking with the Theoretical Domains Framework. And the intervention functions linking to the Theoretical Domains Framework. This figure is from Munir et al.'s published paper [15] adapted from Michie et al.'s work [14]. The inner green circle represents the COM‐B model. The yellow ring in the middle is the Theoretical Domains Framework (TDF) domains linked to each COM‐B category. The red ring includes intervention functions.

The available evidence on participation in VILPA is very nascent. A recent scoping review reported common barriers to participation in VILPA in adults aged 55–75 years were related to a person's skills, while facilitators were related to a person's goals [12]. Social influences and environmental context were found to be both barriers and facilitators to participation in VILPA. One recent study by Thøgersen‐Ntoumani et al. [13] conducted through focus groups explored barriers and facilitators to participation in VILPA in adults aged 36–76. The identified barriers encompassed physical limitations, perception of aging, needing knowledge of VILPA, environmental constraints and fear of injury. Factors that facilitated engagement included convenience, reframing activities as purposeful movement, reminders to participate in VILPA, a sense of achievement, health improvements and self‐reward mechanisms. A strength of Thøgersen‐Ntoumani et al.'s work was the use of COM‐B framework [14] (a behaviour change model that posits a person's behaviour is influenced by the interactions of their capability, opportunity and motivation, see Figure 1). However, the broad age range inclusion may have hindered the authors' ability to thoroughly investigate distinct barriers and facilitators in adults at different life stages. A further limitation of Thøgersen‐Ntoumani et al.'s work was no objective measurements of the participant's own physical activity were made, and this may have made it difficult for the participants to understand the concept of physical activity bout duration and intensity without specific and personal examples to which they could relate. For instance, an individual self‐reporting the participation in a 60 min exercise program may perceive an accumulation of 60 continuous minutes of moderate‐to‐vigorous intensity physical activity [16, 17]. However, the individual's objectively measured physical activity by an accelerometer would consist of bouts of physical activity in light, moderate, and vigorous intensities [16, 17]. Literature also suggests that objective‐measured physical activity reports can increase people's self‐awareness of physical activity behaviours [18].

One of the major life events in adults aged 55–75 is the period of transitioning to retirement. During this time, people may experience life changes such as the loss of income, changing support networks, and change of lifestyle [19, 20, 21]. There is evidence to show that people are more likely to change their behaviour when experiencing major transitions [19, 22]. The likelihood of changing behaviour could be associated with more prioritisation on health [23, 24]. During the transition to retirement period, adults face additional barriers to participation in physical activity such as the personal identity of older age and the association of being less active, change of routine and losing work‐related transportation physical activity, and the onset of multiple health illnesses [23, 24]. Adults transitioning to retirement may also experience unique facilitators to participation in physical activity such as caring for a new grandchild, having more leisure time, and having new goals in life [19, 23]. It is an opportunistic time period in life to intervene in people's physical activity behaviours. Thus, it is important to study how adults transitioning to retirement view their involvement in VILPA so that this information can be used to create a targeted intervention to meet their unique needs. From the authors' scoping review findings [12], a theory‐based semi‐structured guide was developed to explore barriers and facilitators to participation in VILPA. This study aimed (i) to investigate barriers and facilitators from adults transitioning to retirement about increasing physical activity through VILPA, and (ii) to identify potential strategies to promote and implement VILPA.

## 2 Methods

### 2.1 Ethics

Ethical approval for this research was obtained from Curtin University Human Research Ethics Committee (approval number: HRE2022‐0304). All participants gave informed consent for their involvement in the study.

### 2.2 Participants

A total of 46 adults from the general public in the Perth metropolitan area, Western Australia, expressed interest in participating in this study. All 46 adults were screened against the inclusion criteria. Adults were recruited through social media, newspapers, radio advertisements, community public notice boards, and lifestyle villages. The participant inclusion criteria were as follows: (1) adults who were transitioning to retirement, that is, had identified themselves as fully retired in the past 6 months or had plans to retire within the next 5 years (aged 55–75), (2) individuals who were not actively involved in regular exercise (defined as structured exercise programs and sport), (3) those who had not participated in any physical activities causing increased breathing rate (‘huffing and puffing’) for over 10 min in the past week, and (4) individuals who could commit to attending a one‐hour focus group interview. Thirty‐five adults met the selection criteria, and 30 were available and consented to participate. Thirty adults transitioning to retirement, mean age 64.3 years (SD: 4.4; range: 58–72 years), 20 females and 10 males were recruited from the general public in the Perth metropolitan area, Western Australia. Four participants had fully retired in the past 6 months, and the remaining 26 were still working but planning to fully retire in the next 5 years (see Table 1).

**TABLE 1:** Demographic and physical activity data (*n* = 30).

Characteristics			
	Mean	SD	
	*N*	%	

Age (years)	64.3	4.4	
Gender			
Male	10	33.3	
Female	20	66.7	
Employment status			
Full‐time worker	4	13.3	
Part‐time/casual worker	21	70	
Retired (within 6 months)/on leave	5	16.7	
Ethnicity			
White	25	83.3	
Asian	5	16.7	

Physical activity	Median	Range	Mean (standard deviation)
Total vigorous‐intensity physical activity per day (min)	0	0–14.8	1.08 (3.28)
Total moderate‐intensity physical activity per day (min)	35.29	6.5–114.3	44.8 (30.8)
Number of bouts of 5 to 10 min moderate to vigorous intensity physical activity	0.5	0–3	0.77 (0.82)

### 2.3 Procedure

**FIGURE 2:**
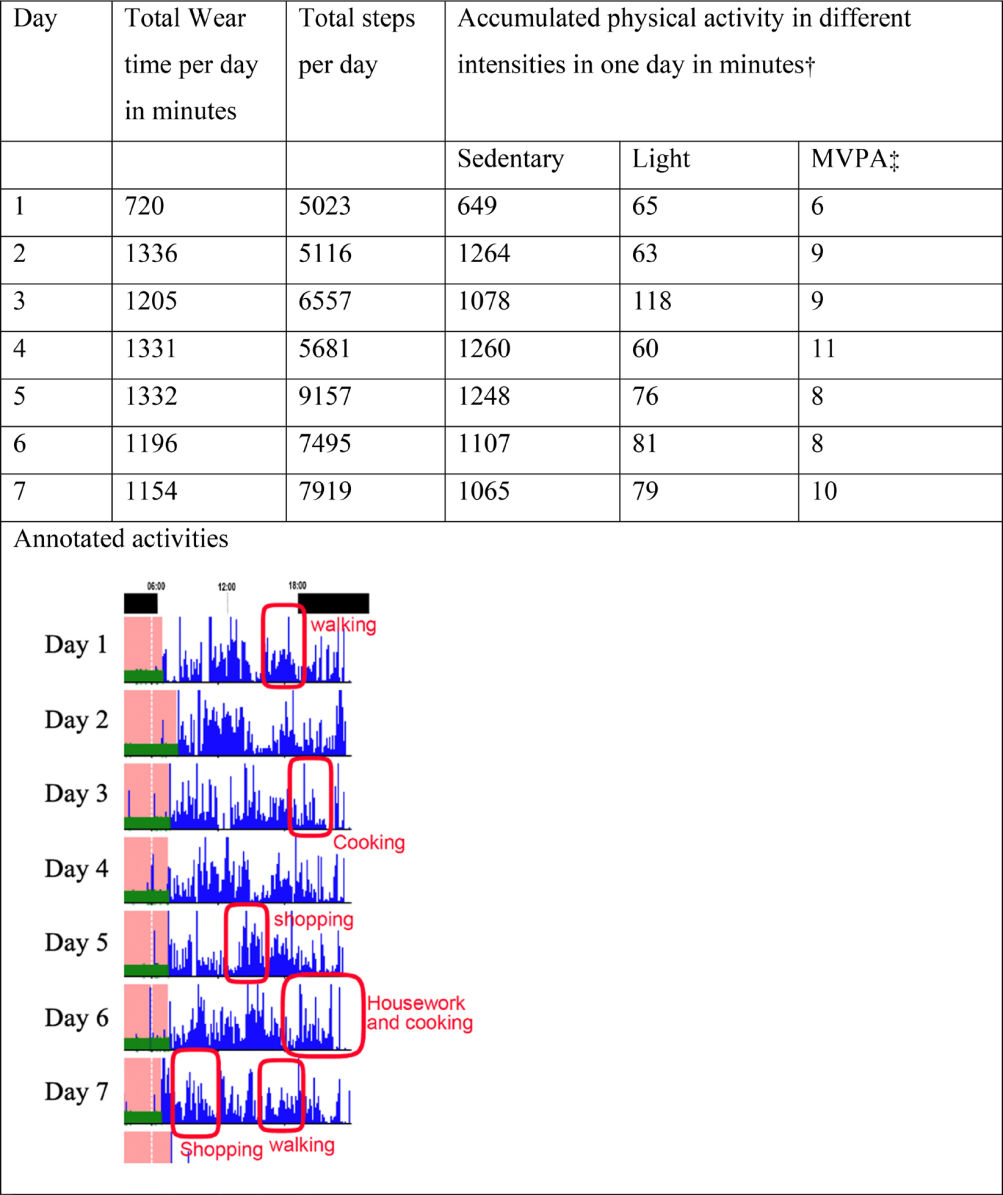
An example of one participant's physical activity findings used prompt discussion in focus groups. †Accumulated physical activity in different intensities were estimated using Montoye cutpoints. ‡MVPA: Moderate to vigorous intensity physical activity.

In the week before the focus group, participants wore an accelerometer (Actigraph GT9X) on their non‐dominant wrist for 7 days and were prompted to complete five Ecological Momentary Assessment (EMA) surveys per day using a smartphone application. Each EMA survey consisted of three questions, including whether the participant engaged in any listed activities (see Table 2) if they were huffing and puffing while doing these activities, and any other activities they did in the past hour. The five EMA surveys were sent at random times to each participant between 8:00 AM and 8:00 PM, and they had 1 h to respond to each survey. Each participant's accelerometer physical activity report was generated using ActiLife version 6 with pre‐set algorithms and cutpoints [25]. The results of the EMA surveys were annotated to the ActiLife‐generated physical activity reports for each participant. Personalised, annotated accelerometer physical activity reports were used to prompt focus group discussions. Figure 2 is an example of one participant's accelerometer physical activity report. The reports were used to aid discussion in the focus group.

**TABLE 2:** Ecological momentary assessment survey.

	Question	Frequency reported^a^
1	In the past hour, did you do any of the following activities?	
	Running for the bus	2
	b Cycling to shops	1
	c Walking to shops	40
	d Carrying shopping for more than 50 m	19
	e Climbing a few flights of stairs	25
	f Looking after or playing with child(ren)	10
	g Mowing lawn	0
	h Vacuuming	10
	i Gardening	39
	j House cleaning	63
	k Sports/exercises	11^b^
	l Lifting or moving heavy objects as a part of my job	7
	m Others	—
2	Were you huffing and puffing while doing the activities?	80
3	Please list the physical activities you have performed which were NOT illustrated in Question 1.	—^c^

^a^ Frequency reported are based on 23 participants who answered at least one ecological momentary assessment (EMA) survey. Two participants could not participate in EMAs because of incompatibility with smartphone devices. Five participants did not answer any EMAs.

^b^ Reported exercises were walking with dog and walking on the beach.

^c^ This was a short answer text answer, the frequency was not counted in this study.

In the focus group, participants were first introduced to the current physical activity guideline recommendations [1] and the concept of physical activity bout duration and intensity. Second, each participant was then provided with a summary and explanation of their individual accelerometer‐derived physical activity findings. Participants were asked to pay attention to their individual annotated physical activity reports of the different intensity patterns of each self‐reported activity. Third, participants were introduced to the concept of VILPA with a figure showing potential types of VILPA (see Supporting Information 1) [1, 4]. A set of semi‐structured focus group questions developed from the findings of the author's previous work (a scoping review [12]) was used to guide the discussions (see Supporting Information 2). The focus group questions were designed to seek deeper insights into the perception of VILPA.

From September to October 2022, a total of three focus groups (approximately 75 min each in duration) with 8 –12 participants in each group were conducted in Perth, Western Australia. Two out of three focus groups were conducted in a hybrid format (i.e., a combination of in‐person and online participation via Microsoft Teams) due to participants' availabilities and health restrictions.

The focus groups were conducted by the first author (female, BPodM MPH, PhD candidate, 10 years of clinical experience working with adults with chronic health conditions). The focus group facilitator had no prior relationship with the participants. The first author wrote reflective notes following each focus group and debriefed with three authors which were used to facilitate the analysis. The participants were compensated with a 50‐dollar supermarket voucher in appreciation of their time.

### 2.4 Analysis

Each focus group was voice‐recorded and transcribed. The transcriptions were imported to and managed in NVivo Version 12. Conventional content analysis was performed by the first author and discussed with another author [26, 27]. All comments and contributions from study participants were treated as equally important, allowing the authors to identify a wide range of intervention strategies. The findings of barriers and facilitators to participation in VILPA were then mapped to the Theoretical Domains Framework (TDF) [28].

The TDF contains 14 psychological domains that influence behaviour (See Figure 1). The TDF is an expansion of the Behaviour Change Wheel (BCW) [14]. The BCW provides a systematic way to understand the barriers and facilitators of physical activity behaviours. The BCW is a synthesis of 19 behaviour change frameworks and consists of the COM‐B model at its core. The capability component of the COM‐B model is expanded to four TDF domains consisting of knowledge, skills, memory and behaviour regulation. The opportunity component is expanded to social influences and environmental context and resource domains. Finally, the motivation component is expanded to reinforcement, emotion, social/professional role and identity, beliefs about capabilities, optimism, intentions, goals and beliefs of consequences of the TDF domains. In this study, the authors inductively mapped the barriers and facilitators to the TDF and then linked them to the COM‐B model. Intervention functions from the BCW methodology were derived from the identified TDF domains using the Behaviour Change taxonomy [14, 28].

Participants' physical activities were screened against the inclusion criteria. To investigate and describe the participation in physical activity of the participants, objective raw accelerometer measured data was further analysed using open‐source R‐package GGIR version 2.9 more comprehensively [29, 30, 31, 32]. Participants with a minimum of 16‐h wear time per day for 7 days were included in the analysis [29, 30]. Accumulated physical activity in different intensities was estimated using Montoye cutpoints [25].

## 3 Results

All participants self‐identified as not participating in regular physical activity or exercises and reported that they had not done any activities that raised their breathing rate for more than 10 min in the past week. During the week of wearing the accelerometer, participants had a median of 35 min of accelerometer‐measured moderate‐intensity physical activity and a mean of 1.08 min of vigorous‐intensity physical activity per day (see Table 1). Twenty‐three participants (76.7%) engaged in the EMA surveys. Of these 23 participants, the average EMA response rate was 60.2% (range = 2.9%–100%, SD = 28%). The most reported activities were walking to shops, house cleaning and gardening (see Table 2). Two participants could not use the EMA smartphone application because of incompatibility with their mobile devices. Five participants did not respond to any EMA survey.

### 3.1 Barriers to Participation in VILPA

#### 3.1.1 Capability

##### 3.1.1.1 Deteriorating Physical Health and Chronic Health Conditions

Participants reported feeling physically unable to engage in some forms of VILPA. For instance, a few participants reported difficulties climbing a few flights of stairs because of knee and hip conditions. ‘You don't see old people climbing stairs … depending on the rate’. ‘… my knees stop me from climbing stairs …’ ‘Stairs is an issue for me … I have multifocal glasses …’ In addition, some participants reported having cardiorespiratory conditions, and they were afraid of cardiorespiratory adverse events if their heart rates were ‘too high’.

##### 3.1.1.2 Lack of Knowledge of VILPA

As VILPA is a new concept, adults transitioning to retirement felt they lacked understanding of the meaning and feeling of intense daily physical activities. ‘I need to know how this intensity feels like’. and ‘What do you mean by increasing intensity. That's what I need to understand’.

#### 3.1.2 Opportunity

##### 3.1.2.1 Extreme Weather Conditions Affect VILPA Participation

Participants reported poor weather would affect any activities that are performed outdoors. ‘Too hot or too cold… I don't want to go outside’. ‘Walking when it's windy, I don't like it’. Neighbourhoods with low walkability were also reported to affect participants willingness to engage in VILPA, as a participant reported ‘My area has uneven footpaths, I don't feel safe to walk outside when it gets darker’.

##### 3.1.2.2 Lack of Support

Participants reported difficulties finding people to do VILPA with even if they wanted to, ‘I don't know anyone in my community … to do the activities together’. In the transitioning to retirement period, participants reported facing difficulties in engaging in activities together with friends and family, one participant said ‘It's hard to coordinate things with my friends. We work on different days,’ another participant reported ‘I used to play sports with my son, now he's grown up… you know… we don't really get around to do that….’

#### 3.1.3 Motivation

##### 3.1.3.1 Fear of Injury

Participants in this study reported feeling unmotivated to engage in vigorous physical activities because of fear of injuries. ‘You take longer to heal when you are older. And you fear about to injuring yourself’. Although they may be physically able to perform VILPA such as stair climbing, some participants were not confident to do so, ‘I had that feeling of falling… going up (stairs) is ok, coming down is difficult’.

##### 3.1.3.2 Self‐Perceived Lack of Time

The participants felt they did not have time to engage in any physical activities because of long working hours. Especially adults who were still working full time, ‘I've worked a lot of 12‐hour shifts in the last couple years, so exercise has been a real issue. I can't seem to fit it in’. Adults who are recently retired felt they were not able to participate in physical activities as they were not able to fit it in with retirement plans. ‘I am going away for six weeks, and at times, you know caravanning… most of the time we'll be sitting in a car’.

### 3.2 Facilitators to Participation in VILPA

#### 3.2.1 Capability

##### 3.2.1.1 Understanding of VILPA

Participants felt that by knowing more about VILPA they would participate. ‘… knowing that this activity is going to give me x minutes I need (each day), but you have to have that knowledge to go on with’. As each person may have different health conditions, ‘knowing that tiny bit can make it intense, and know what it feel like would make a huge difference’. In addition to the intensity, ‘knowing how much VPA you need is important’, as it would help adults transitioning to retirement to plan their VILPA each day.

##### 3.2.1.2 Feasibility of VILPAs

Participants reported the accumulation of VILPA is ‘easy’ as they are able to integrate the activities into their schedule. ‘Is 11 minutes the whole day? Is that all? Wow that's pretty doable’. Participants also felt that the examples of VILPA were acceptable and were activities they were already doing in their life. ‘I'm sure I can do all the activities’. ‘I can do all these things… I go to a physio occasionally for a back problem but that's about it’.

##### 3.2.1.3 Monitoring of VILPA

Adults transitioning to retirement felt that regular reporting of their VILPA engagement would be beneficial. ‘If you were to report in some way, daily … that would make me more inclined to do it’. Participants felt it would be useful to track their daily engagement in VILPA themselves, to ensure they meet their weekly goals.

#### 3.2.2 Opportunity

##### 3.2.2.1 Support From People in Similar Life Stage

Having support from family and friends would motivate adults transitioning to retirement to engage in more VILPA. ‘I think like if you have a friend or something doing the same too … You can do these things together when you meet up you know …’ Participants also feel that knowing a group of people who also engage in a VILPA program would be helpful as ‘Doing things in a group is easier than on your own’.

#### 3.2.3 Motivation

##### 3.2.3.1 Being Fit for Retirement

Participants reported that they were willing to engage in VILPA and perceived physical activity as important. As their age increases, participants reported declining health as a major challenge and would like to maintain their fitness. ‘I'm looking forward to retirement but I'm not looking forward to not being fit for retirement … I want to continue with my (motor)biking and fishing, those things really need my fitness’.

Maintaining physical health to do things they enjoy. ‘I dropped my bike the other day … and I couldn't pick it up I used to be able to do that six months ago. I need to do something about it’. Participants reported wanting to engage in more hobbies when they retire, and felt increasing physical activity would be beneficial to maintain their physical health. ‘I need that muscle strength and want to do other things, I love fishing’. ‘I probably would like to increase my physical fitness so that after one or two dances I am not puffing…’ ‘…if you are travelling, you wanna go for a hike, you don't wanna be puffing and panting…’.

##### 3.2.3.2 Positive Feedback on Achievements

Participants reported feeling more motivated knowing they have reached the appropriate intensity when doing VILPA and achieved their daily targets. ‘Something that shows the intensity on the feedback (would help me)’. ‘Something to tell me I have done my x minutes today… something gives you a report’.

##### 3.2.3.3 Negative Emotions Toward Vigorous Physical Activity

Participants reported experiences of exercise programs at local gyms and did not enjoy the environment of gyms. ‘I don't like going to gyms. I just don't go anymore’. Adults transitioning to retirement feel that they do not feel they belong to the gym. On the other hand, VILPA offers micro‐bouts of activities which is more attractive than attending exercises at the gym.

### 3.3 Intervention Functions

To identify intervention functions that could be used to promote and implement VILPA, barriers and facilitators were mapped to the TDF domains. The barriers mapped to seven TDF domains (knowledge, skills, environmental context and resources, emotion, social/professional role and identity, and beliefs about consequences), and the facilitators mapped to 11 TDF domains (knowledge, behavioural regulation, skills, social influences, environmental context and resources, reinforcement, emotion, social/professional role and identity, belief about capabilities, goals and beliefs about consequences). Intervention functions derived from the TDF domains were education, persuasion, incentivisation, environmental restructuring, modelling and enablement (see Table 3).

**TABLE 3:** Barriers and facilitators to participation in VILPA in adults transitioning to retirement. Barriers and facilitators were mapped to the Theoretical domains framework (TDF), linked to the COM‐B model and intervention functions of the Behaviour Change Wheel methodology.

COM‐B dimension	TDF domain	Codes	Themes	Illustrative	Intervention functions
Barriers					
Capability	Physical skills	Health conditions, pain	Age‐related physical limitations	‘My body changes as I age. I can see the signs of aging. I don't have the strength to do the same thing as before’. ‘I know the differences in my health before I got diagnosed with glaucoma’. ‘A four‐year‐old is very heavy. I got an arthritic shoulder. I don't have the strength to carry them. I got pain in my foot so I find that I can't walk very far. But I put up with it, it is not good. So that puts me off… My knees are troubling. I can't climb stairs for sure’.	Education Training
	Knowledge	Uncertain about intensity	Need for more knowledge	‘I need to know how high this high intensity is. What is it for me? I think I need to know what it feels like, that is important. So I know how hard I need to go’.	Education
Opportunity	Social influences	Lack of active social opportunities	Need for social support	‘It's hard to coordinate things with my family or friends. We work on different days and I can only do things myself’. ‘I have no one to play sports or walk with. My children are all grown up, we don't get around to do things together anymore’.	Environmental restructuring Modelling
	Environmental context and resources	Poor weather, living location	Temperature constraints Living environmental constraints	‘When it is too hot or too cold outside. Even when it is too windy. It's unpleasant’. ‘A lot of footpaths around my neighbourhood is uneven. I wouldn't walk around there’. ‘I don't have garden or lawn at my place. So that wouldn't be applicable to me… I don't have young children or grandchildren’.	Environmental restructuring Modelling
Motivation	Social/professional role and identity	Self‐identity	Perception of self and others	‘I don't see anyone my age taking stairs. I see everyone drives’.	Modelling
	Beliefs about consequences	Fear of injury Self‐perceived lack of time	Fear Lack of time	‘I think with stairs I need to be careful. Going up is ok, but coming down… I need to be careful. I hurt myself a few years ago’. ‘I don't know how much is too much. I don't want to hurt myself you know’. ‘I leave home at 7.30 in the morning, drive to work, get home and sitting at a computer all day for 8 h, then get home about six o'clock. So I just wonder how I can sort of put any of these in and we don't have stairs at work. I'm basically sitting at a computer is like, well, when do I get to sort of do this kind of thing?’	Education Persuasion Modelling Enablement
Facilitators					
Capability	Physical skills	Flexible in duration of activities	Accumulation of physical activity bouts	‘I can start slow and build up my minutes’. ‘You can make up your own “exercise” and see what suits yourself the best’.	Education Training
	Knowledge	A better understanding of VILPA and intensity	Understanding of VILPA	‘Having something specific so we know which activities can be VILPA would be good’.	Education
	Behaviour regulation	Self‐recording of activities performed	Monitoring and tracking	‘I want to know how I can record my activity minutes, like which type of activities I can do to benefit me. That would help a lot’.	Education Enablement
Opportunity	Social influences	Advises by health professionals	Professional influences	‘I found helped me a lot was having regular medical check‐ups and getting on top of those things like my doctor said to me’.	Modelling Enablement
		Support from family and friends	Social support	‘Doing things in a group is easier than on your own. So if you've got a set time with a friend how to get out that's a commitment’.	
	Environmental context and resources	Pleasant weather, safe environments, pet ownership	Conducive environments	‘Fresh air. Nice when you are out in the fresh air doing things’. ‘For me, it's knowing there will be handrails if it's going on the stairs’. ‘If you have a pet, that will get you walking outside’.	Environmental restructuring Enablement
Motivation	Social/professional role and identity	Setting an example for others	Identify being healthy and active	‘Well, I try to set an example. We all take an example, to get everyone going. So, I would want to set an example for others’.	Modelling
	Beliefs about capabilities	VILPA is easy to accumulate, acceptable	Achievable targets	‘Those are pretty achievable to me. Not a high target… I can do all those activities, it's not a problem’. ‘Brisk walking is very acceptable. I got 10 min, I can brisk walk around the block’.	Education Enablement
	Goals	Goal to live longer, be active and fit, focus on self	Maintain health and fitness	‘My mother lived til her 90s, I want to get there too. I want to maintain some strength, my joints, and muscles. Maintain muscle strength and do other things I like. I love fishing’. ‘I'd like to start to focus on where I'm going (being more active), personally’.	Persuasion Incentivisation Enablement
		Health benefits	Avoid chronic health conditions	‘Keep myself active so I don't get health issues. I want to lose some weight’.	Incentivisation
	Beliefs about consequences	Health complications	Deteriorating health	‘I'm looking forward to retirement but I'm not looking forward to not being fit for retirement’. ‘I have a lot of free time, but I don't do anything. It's not good for my health’.	Persuasion Modelling
	Reinforcement	Notice on achieving VILPA	Feedback on achievement	‘Perhaps getting a report or some sort of feedback to let me know I've done VILPA every day’. ‘Maybe a rewards system. It doesn't need to be money. I just need to know I have done it’.	Incentivisation Environmental restructuring
	Emotion	Positive feelings towards doing VILPA	Positive emotions	‘I feel like that I will be doing something benefits me. Psychologically, I feel good. It means I am able to do those things without any fear’.	Persuasion Enablement
		Negative previous exercise experiences	Previous experiences associated with gyms	‘I don't like going to the gym and do workouts. I don't feel like I fit it. Those things in the gyms are not for me’.	Enablement

## 4 Discussion

The perspectives of adults transitioning to retirement are that engaging in VILPA holds promise for sustaining physical well‐being and represents a viable avenue to enhance their overall physical activity levels. Obstacles to participation in VILPA stem from unfavourable attitudes toward vigorous physical activities, health constraints and physical limitations associated with aging, insufficient awareness about VILPA, and challenges posed by adverse weather conditions. To promote participation in VILPA, adults transitioning to retirement require a better understanding of physical activity intensities, knowledge about how to identify VILPA opportunities, monitoring VILPA engagement, social support and feedback for achievements. The findings from this study may contribute to the development of targeted and personalised interventions aimed at fostering short bouts of physical activity such as VILPA in adults transitioning to retirement.

The perceptions of participation in VILPA in adults transitioning to retirement in the current study have some similarities and differences compared with previous studies [12, 13]. Although other studies have reported that adults perceive older age as a barrier to participation in vigorous‐intensity physical activity, participants in this study reported no issues if they were to engage in most types of VILPA presented during the focus group [13, 33]. VILPA consists of performing activities that already exist in an individual's daily life with more vigour. Thus, adults transitioning to retirement may perceive VILPA as being more acceptable to perform when compared with structured vigorous‐intensity exercises that require additional equipment and skills to perform. Each VILPA bout may only last ≤ 2 min, and as such participants of older age may perceive short bouts of vigorous‐intensity physical activity as being more achievable than sustained longer durations of structured exercises. Climbing a few flights of stairs was identified as challenging for adults with knee‐related musculoskeletal conditions, however, participants in this study felt they could still be physically able to engage in other options of VILPA. Like other studies, environmental constraints were revealed as a key barrier. Unlike previous findings that highlighted limited access to stairs or hills in the neighbourhood, the primary environmental hindrance in this study revolved around poor weather conditions [13, 34]. Such differences are likely to be because of some forms of VILPA being performed outdoors that can be affected by poor weather conditions, for example, carrying shopping for ≥ 50 m, brisk walking to the shops, lawn mowing and gardening.

A range of facilitators were reported in this study. One facilitator for adults transitioning to retirement to engage in VILPA is the augmentation of knowledge about VILPA, similar to Thøgersen‐Ntoumani et al.'s work [13]. As VILPA is a new concept and it differs from existing structured vigorous‐intensity exercises, study participants emphasised the first step to facilitate participation in VILPA is knowing their own vigorous intensity level and using their own vigorous intensity level as a reference point to engage in potential VILPA in their daily lives. Study participants conveyed that their individualised accelerometer report provided valuable insights into the accumulation of physical activity and an understanding of activity intensities. Their reports contributed to their improved comprehension of VILPA. In addition to tracking activity, establishing personal thresholds for vigorous intensity aids in developing a deeper awareness of what vigorous‐intensity activity entails. Moreover, a comprehensive understanding of self‐monitoring mechanisms and their impact on fostering healthy habits further bolsters the inclination toward VILPA participation.

## 5 Implications

The results of this study indicate the feasibility of increased physical activity participation through VILPA among adults transitioning to retirement. For the demographic of participants in the current study, the accumulation of VILPA appears to be an attractive option. Barriers and facilitators to participation in VILPA were identified across different TDF domains, therefore multiple‐pronged approach using a range of intervention functions would be ideal in a targeted intervention. Future interventions to promote VILPA could consider strategies that utilise a combination of education, persuasion, incentivisation, environmental restructuring, modelling and enablement. A combination of the six strategies will ensure that all components of the COM‐B model are addressed. For instance, increasing an individual's knowledge about the health benefits of short bouts of vigorous‐intensity physical activities, utilising existing functional fitness tests to seek an individual's vigorous intensity level while incorporating the recently developed empirical definition of VILPA, having regular reminders and encouragement to initiate health behaviours, assist individuals in setting achievable goals, and providing a community to support individuals to be more physically active [35, 36]. The current findings could facilitate the development of a targeted intervention for adults transitioning to retirement. Future research could involve key stakeholders such as the target population (adults transitioning to retirement) and professionals with experience in physical activity promotion during the development and implementation of the intervention to ensure the intervention contents and implementation strategies are acceptable, practical, effective and affordable.

Intervention strategies such as regular prompts and reminders and ongoing monitoring could potentially be implemented via a smartphone application. Just‐in‐time adaptive intervention (JITAI) assists in the delivery of intervention prompts at the ‘right time’ and the ‘right place’, however, it may be challenging in older adults because of lower smartphone literacy [37]. Adults transitioning to retirement should be consulted in regard to the mode in which an intervention is to be delivered to ensure it is appropriate.

Some retiring adults may require the support of a health professional because of chronic health conditions and fear of ‘doing the wrong thing’. For instance, a retiring adult could gain reassurance from their family doctor that participation in VILPA is aligned with their health conditions management goals. Health professional support could also be beneficial for ongoing monitoring and tracking of an individual's progress. Adults transitioning to retirement may experience several chronic health conditions and under the care of different health professionals. Hence, a range of health professionals with experience in promoting physical activity and managing adults with chronic health conditions should be consulted in developing and implementing a targeted VILPA intervention. A recent consensus statement also substantiates the multidisciplinary approach to improving physical activity behaviours [38]. We recommend intervention designers consult with a range of different health professionals to ensure the intervention's applicability, feasibility and appropriateness in various clinical settings. This will also assist with the future dissemination of the intervention.

### 5.1 Strengths and Limitations

This is the first mixed‐method study that combines an intervention development theory with physical activity data to explore barriers and facilitators to participation in VILPA in adults transitioning to retirement. The incorporation of previous scoping review findings and the TDF ensured the focus group questions were crafted with specific objectives to investigate the barriers and facilitators in the specific target population for the future development of an intervention. Personalised and annotated physical activity data used in the focus group facilitated a clearer understanding of the intensity and bout duration of VILPA in the participants.

There are some limitations of this study that should be taken into consideration in the interpretation of the findings. This study was conducted in the Perth metropolitan area in Western Australia. It is an urban city where the majority of the infrastructure is designed to accommodate vehicles, making it difficult to do active commutes (e.g., walking and cycling as a form of transport). Barriers identified in this study such as inconducive environments are confined to the specific city landscape and may not be generalisable to regional settings. Location‐specific adjustments should be considered when promoting VILPA outside of metropolitan areas. Study participants' self‐reported physical activity and accelerometer‐measured physical activity findings should be interpreted with caution. Although all participants in this study self‐reported not engaging in regular physical activity or exercises, a group of participants with a median of 35 min of moderate‐intensity physical activity per day (measured by wrist‐worn accelerometer) were inadvertently recruited in this study. Each participant was screened via telephone before enrolment into the study to ensure that they were not participating in regular exercises. Participants were also instructed to continue their usual routine while wearing an accelerometer. It is possible that the moderate‐intensity physical activity findings were associated with non‐structured activities or exercises. It is also possible that the accelerometer wear generated reactivity in the participants, which led to the presentation of increased accumulation in moderate‐intensity activity [39]. In this study, wrist‐worn accelerometers were used which also tends to overestimate physical activity levels [40]. Future research to assess intervention effects in promoting VILPA should have multiple accelerometer measurements to address the changes in physical activity compared with baseline. Participants in this study were interested to learn more options to increase their physical activity which may have influenced their positive attitudes toward VILPA. Future research to develop and implement a VILPA intervention should consult a range of health professionals with experience in promoting physical activity and managing chronic health conditions in adults who may be less hesitant or motivated to change their physical activity behaviours. Such health professionals could provide strategies to persuade individuals to change their physical activity behaviours in clinical settings.

## 6 Conclusion

Adults transitioning to retirement perceive participation in VILPA as both feasible and convenient for increasing their overall physical activity levels. Multiple barriers and facilitators were identified, and these should be addressed and utilised, respectively, to develop targeted interventions and associated implementation plans. To develop effective interventions, collaboration with health professionals experienced in promoting physical activity is recommended, along with input from adults transitioning to retirement to ensure implementation feasibility and relevance.

## Ethics Statement

Ethical approval for this research was obtained from Curtin University Human Research Ethics Committee (approval number: HRE2022‐0304). All participants gave informed consent for their involvement in the study. All procedures performed in studies involving human participants were in accordance with the ethical standards of the institutional and/or national research committee and with the 1964 Helsinki Declaration and its later amendments or comparable ethical standards.

## Consent

Informed consent was obtained from all individual participants included in the study.

## Conflicts of Interest

The authors declare no conflicts of interest.

## Supporting information


Data S1.

## Data Availability

The data that support the findings of this study are available on request from the corresponding author. The data are not publicly available due to privacy or ethical restrictions.
