# Supplementary material for: An Exploration of Adults Transitioning Into Retirements' Perspectives on Vigorous Intermittent Lifestyle Physical Activity
Source: Health Promot J Austr. 2025 Jan 7;36(1):e957. doi: 10.1002/hpja.957 (PMC11739672; doi:10.1002/hpja.957)
Supplement: Supplementary file 1 — Data S1. [file HPJA-36-0-s001.pdf]

Supplementary material 1

Examples and accumulation of VILPA

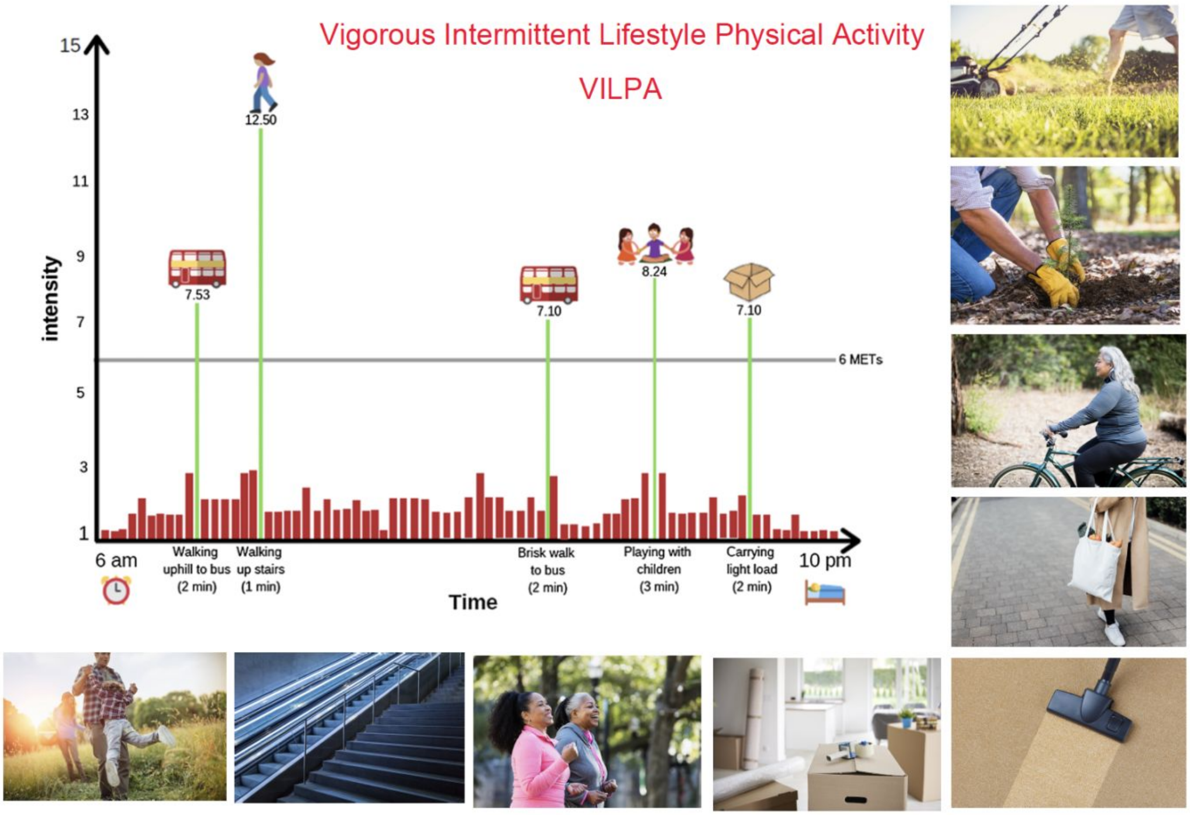

Supplementary material 2: Adults transitioning to retirement focus group guide.†

	**Barriers**
**TDF domain**	**Semi-structured focus group stimulator questions**
Skills	Tell me about your physical health, how is your body and how well are you able to do daily activities? Such as climbing a flight of stairs, playing with, or carrying a child, carrying shopping for more than 50 metres, mowing the lawn, and planting pot plants.<br>How healthy / fit do you feel in doing these daily activities? Such as climbing a flight of stairs, playing with, or carrying a child, carrying shopping for more than 50 metres, mowing the lawn, and planting pot plants.<br>Are there any health conditions stopping you from doing these more intense daily movements?
Beliefs and capabilities	How do you think you would find doing VILPA?<br>Do you see yourself as someone who would do VILPA every day?
Social / professional role and identity	I will name some daily activities, and each time, I want you to raise your hand if you think doing these activities is accepted among your age group? (raise hands / elaborate)<br>Brisk walking, Climbing stairs, Carrying a small load of shopping, Carrying or playing with a child, Gardening, Housework
Environmental context and resources	Are there any environmental or other factors that would stop you from doing VILPA? E.g., house location, pets, grandchildren, availability of parks / shopping centre parking / usage of shopping bags
Beliefs and consequences	What harm or consequences do you see there may be to your body if you were to do VILPA such as the examples provided? Such as climbing a flight of stairs, playing with, or carrying a child, carrying shopping for more than 50 metres, mowing the lawn, and planting pot plants.
Others	What else would prevent you from doing VILPA?

	**Facilitators**
**TDF domain**	**Semi-structured focus group stimulator questions**
Social influences	How would social support from either family or friends influence you doing VILPA?

Environmental context and resources	Are there any environmental factors that would encourage you to do VILPA daily?
Social / professional role and identity	Do you see yourself as someone who would do VILPA every day? What other activities do you do usually in your day, that is not a form of exercise, but gets your 'huffing and puffing'?
Goals	Are there any physical fitness or health goals you would like to achieve during this time period of yours?<br>What sort of goals do you think are appropriate?
Others	What else would encourage you to do VILPA every day?<br>Comments…

†Questions derived from the scoping review.
